# Symmetry of computerised tomography of the brain in traumatic brain injury: a quality improvement audit

**DOI:** 10.1186/s12883-023-03441-x

**Published:** 2023-10-31

**Authors:** Ajish Sam George, Pragnitha Chitteti, Shalini Nair, Reka Karuppasami, Mathew Joseph

**Affiliations:** Christian Medical College, Vellore, Tamil Nadu India

**Keywords:** CT symmetry, Laser lines, Head position, Checklist

## Abstract

**Background:**

Non-contrast Computerised Tomography (NCCT) of brain is the gold standard investigation for diagnosis and management of Traumatic brain injury (TBI). Asymmetrical CT brain images as a result of improper head positioning in the CT gantry will compromise the diagnostic value. Therefore, this audit aimed to assess the degree of asymmetry in CT brain studies carried out in TBI patients.

**Methods:**

This audit was carried out at a level one trauma centre and included CT scans of TBI patients with a Glasgow come scale (GCS) score ≤ 13, admitted to the Neurological intensive care unit (NICU). The first cycle involved a period of three months. The data collected included demographic data and variables such as GCS at the time of the scan and whether the patient was intubated or not. The visualisation of bilateral internal auditory meatuses was used as landmark to determine scan symmetry. If the internal auditory meatus on both sides were visible on the same slice of CT scan, it was considered symmetric. The degree of asymmetry was gauged based on the axial slice difference between bilateral meatuses. The data collected was tabulated and presented to Neurosurgery residents and a checklist was formulated which had to be followed while positioning the patient on CT table prior to imaging.

**Results:**

The first cycle of the audit showed that 83.8% of scans were asymmetric and among them 44.1% revealed gross asymmetry affecting interpretation of the scan. Following, implementation of the checklist the percentage of gross asymmetry dropped to 21.86% in the second and to 22.22% in the third audit.

**Conclusion:**

The use of checklist prior to CT brain studies showed sustainable improvement in reducing gross asymmetry and in acquisition of symmetrical CT brain images.

**Supplementary Information:**

The online version contains supplementary material available at 10.1186/s12883-023-03441-x.

## Key message

• What is already known on this topic –

The importance of symmetry in CT scan slices of brain is known to be vital in interpreting the image. In TBI multiple causes lead to asymmetry in this vital diagnostic tool.

• What this study adds –

We found the magnitude of the problem to be very significant (83%). The cause of asymmetry was predominantly head positioning due to lack of attention to this step.

An application of checklist ensuring correct head positioning not only reduced the asymmetry, but was also found to be sustainable measure.

• How this study might affect research, practice or policy – This study will help reduce asymmetry in NCCT, thereby, increasing the value of this basic test.

**Supplementary Information:**

The online version contains supplementary material available at 10.1186/s12883-023-03441-x.

## Background

Non contrast computerized scan of brain is the gold standard for management of TBI. However, asymmetrical CT slices make interpretation difficult, thereby reducing the value of this test. It is a problem faced regularly in Neurological ICU (NICU). Hence we decided to collect data to estimate the extent of the problem and attempt to resolve it. Traumatic brain injury (TBI) is predicted to be one of leading causes of morbidity and mortality in India [1, 2]. Establishment, adherence and review of protocol based investigative approach is key in managing head injury. Non-contrast Computerised Tomographic (NCCT) scan of brain is the gold standard investigation for diagnosis and management of TBI [3, 4]. Interpretation of central nervous system (CNS) anatomy is aided by the fact that the brain’s two halves are nearly symmetrical. Asymmetrical acquisition of CT brain detracts from the quality of information obtained from the scan and will reduce the diagnostic value of the study [5]. Moreover, CT symmetry helps the interpreter to compare two sides of the brain and diagnose cranial pathologies [6].  Unfortunately, most of the CT brain scans done in emergencies have varying degrees of asymmetry [7]. There are a number of reasons which contribute to asymmetrical CT brain scans such as improper head positioning, agitated or non-co-operative patients or inexperience of the person positioning the patient [8]. Therefore, this audit aims to assess the degree of asymmetry in CT brain imaging done in TBI patients.

### Measurement

#### Study population and data collection

The audit was conducted in a level one trauma centre in the state of Tamil Nadu, India. In this audit we included non-contrast computed tomography (NCCT) scans of all admitted head injury patients with a GCS score ≤ 13 (our protocol mandates that these patients be accompanied by a Neurosurgery resident). Information collected included patient-specific data such as age, gender, date of scan, diagnosis, degree of symmetry in terms of axial CT slice difference between bilateral internal auditory meatuses, GCS at the time of the scan and airway status (intubated or not) (supplementary material).

As it was a quality improvement initiative, need for ethics approval and informed consent was waived by the Institutional Review Board of Christian Medical College, Vellore, India.

#### Inclusion criteria

TBI patients with a GCS score ≤ 13, accompanied by Neurosurgery residents for the scan and admitted to the Neurosurgical ICU.

#### Exclusion criteria

Patients who were not accompanied by a Neurosurgery resident to the CT room were excluded (occasionally the patient would be escorted by the Emergency Department physicians).

TBI patients not admitted in our hospital after CT scan were also excluded.

#### Standards set and rationale

**Fig. 1:**
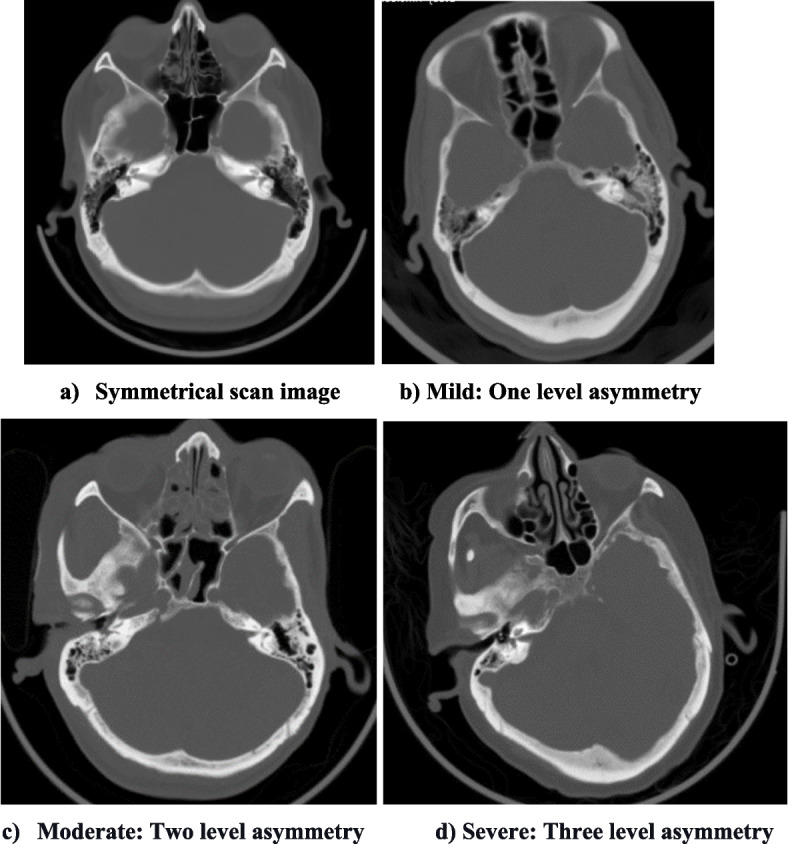
Representative images of symmetrical and various degrees of asymmetry. **a** symmetrical scan image. **b** mild: one level asymmetry. **c** moderate: two level asymmetry. **d** severe: three level asymmetry

On a normal CT brain, the two halves of the brain are mirror images. Since the two sides are comparable, detection of abnormality is easier. However if, the CT image is acquired with the head tilted, this symmetry no longer exists. Hence, interpretation becomes more difficult. To gauge the symmetry in CT brain we need to compare simultaneous visualisation of homologous structures in the same axial CT slice (Fig. 1). In this audit, we considered visualisation of bilateral internal auditory meatuses in the same axial slice (thickness 5 mm) of the CT brain image as a marker of a symmetrical scan. This method of assessment was quick, objective and straightforward without the use of any sophisticated algorithm.

#### Strategy

**Fig. 2:**
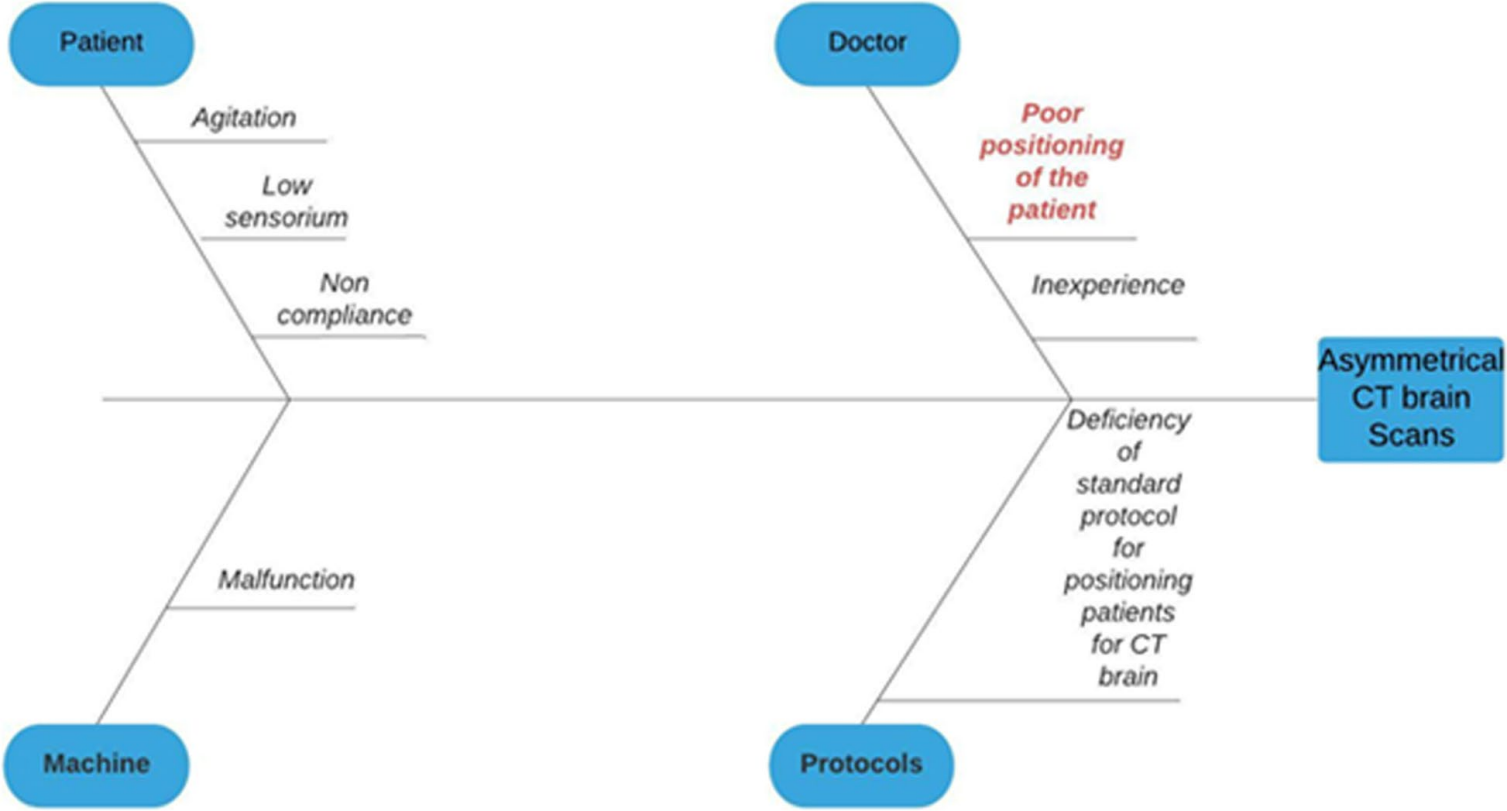
Fishbone analysis of causative factors for CT Head asymmetry

A lapse in quality is most likely to be multifactorial in origin. To rightly identify the probable causes contributing to the problem (asymmetrical CT scans), we systematically analyzed and categorized the causes into different domains using a fish-bone analysis [9] (Fig. 2).

#### Design

Two principal investigators analysed CT brain imaging in TBI and assessed the symmetry of the scans from our prospectively collected patient data base. The degree of asymmetry was categorized by number of slices by which the two internal auditory meatuses are separated into the following categories: mild (one slice difference), moderate (two slices difference) and severe asymmetry (3 or more slices). In cases of disagreement, consensus was reached after review of CT scan by the seniormost author (MJ) and his decision was accepted. Both moderate and severe symmetrical scans were categorized as gross asymmetry, significantly affecting interpretation. The data was analyzed, and factors contributing to asymmetrical CT brain scans were identified.

From this analysis we identified and reached a consensus that poor positioning of patient’s head was major cause for the problem. An intervention in the form of a CT Brain checklist (supplementary material) was implemented, which had to be completed by doctor accompanying the patient. The checklist included checkboxes with the following steps (to be completed while positioning the patient on CT table).

**Fig. 3:**
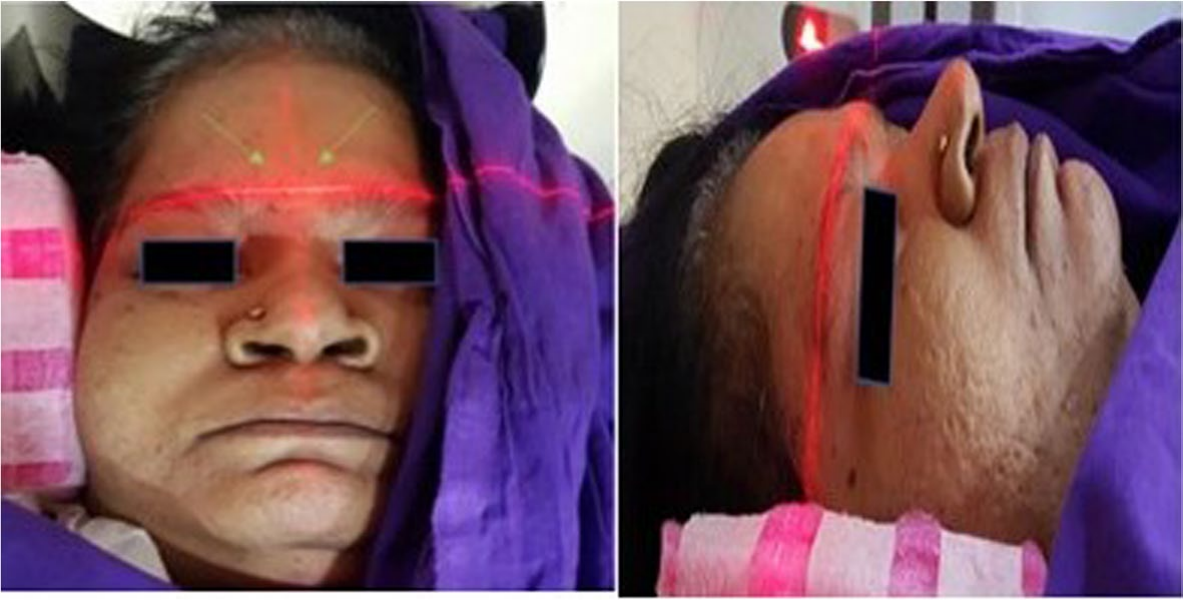
Figure illustrating correct head positioning using laser lines

1. Ensure horizontal and vertical Laser marker lines of the CT machine intersect at the Glabella (Fig. 3).
2. Horizontal marker line should superimpose over the supraorbital ridges bilaterally, and the vertical marker line should be in the midline, and both the lines should intersect.
3. The patient should be immobile.
4. Ensure safe and adequate sedation if needed.

#### Statistical analysis

The number of patients and percentages were presented for categorical data. The Chi-square test was applied to find the association between categorical variables. All tests were two-sided at α = 0.05 level of significance. All analyses were done using Statistical Package for Social Sciences (SPSS) software Version 21.0 (Armonk, NY: IBM Corp).

## Results

### Baseline measurements

The first cycle of the audit was conducted for four months, from November 2019 to February 2020. It revealed that the majority of CT scans (83.8%) were asymmetric. 39.7% of scans showed only minimal asymmetry with a difference of 1 slice, but the remaining 44.1% were asymmetric enough to cause difficulty in interpretation (Table 1).

**Table 1:** Comparison of severity of asymmetry before and after intervention

Level of internal auditory meatus	Pre-intervention symmetry of the CT scan	Post intervention symmetry of CT scan	Post intervention sustenance of symmetry of CT scan
Same CT slice	11 (16.2%)	22(30.14%)	26 (39.39%)
Minimal asymmetry - Difference of 1 slice	27 (39.7%)	35 (47.95%)	26 (39.39%)
Gross asymmetry - Difference of 2 slice	14 (20.6%)	12 (16.43%)	6 (9.10%)
Gross asymmetry - Difference of 3 slices	16 (23.5%)	4 (5.43%)	8 (12.12%)
Total	68	73	66

These were mainly in patients who were not intubated, which probably contributed to a higher degree of asymmetry (Table 2). However, even among the 31 intubated patients who were sedated, only 19.35% of the scans were symmetric, implying a lack of attention to correct positioning of the patient’s head in the CT gantry. Therefore, it was apparent that significant improvements were needed in ensuring a symmetrical CT brain scan with good diagnostic value.

**Table 2:** Comparison of airway status between patients with symmetrical and asymmetrical scans

	Cycle I				Cycle II				Cycle III			
	Symmetric n (%)	Asymmetric n (%)	Total n	*P* value	Symmetric n (%)	Asymmetric n (%)	Total n	*P* value	Symmetric n (%)	Asymmetric n (%)	Total n	*P* value
Not intubated n(%)	5 (13.5%)	32 (86.5%)	37	0.5147	17 (33.33%)	34 (66.67%)	51	0.3648	14 (40%)	21 (60%)	35	0.9147
Intubated n (%)	6 (19.35%)	25 (80.65%)	31		5 (22.7%)	17 (77.3%)	22		12(38.71%)	19 (61.29%)	31	
Total n (%)	11 (16.2%)	57 (83.8%)	68		22 (30.14)	51 (69.86)	73		26 (39.4%)	40(60.6%)	66	

Subsequently, doctors accompanying the patient were alerted and advised to follow the checklist (supplementary material) and the steps therein, before repeating the audit cycle. Following the intervention, percentage of grossly asymmetrical scans dropped from 44.1 to 21.86%. In addition, the percentage of symmetrical scans almost doubled in comparison to the first audit from 16.2 to 30.14% (Table 1).

A third cycle of the audit was undertaken to ensure the sustainability of the intervention. This made it clear that the intervention was effective as the percentage of grossly asymmetric scans dropped from 44.1% in the first cycle to 21.22%. Moreover, the percentage of perfectly symmetrical scans increased from 16.2% in the first audit to 39.39% with the third cycle (Table 1).

The effect of contributing factors as severity of head injury or intubation status was not significant (Table 2).

## Discussion

Head injury has a complex and varied presentation making it difficult to manage. Over the years head injury assessment and management has evolved with the use of standardised protocols that has helped improve treatment outcome. Symmetrical CT scans of the brain are an integral part of management in TBI. Unfortunately, in our high volume centre majority of NCCT of head injury patients were asymmetric affecting their diagnostic value. Confounding factors like agitation or sedation were ruled out as the effect of intubation in all 3 cycles with any severity of head injury was not statistically significant.

The use of checklist with objective parameters using laser beam helped attain correct position effectively. A good checklist enables medical providers to work more efficiently by serving as cognitive aid in high intense environment requiring multitasking and also prevents inter-physician variability [10, 11]. In addition, the use of checklist in health care helps to improve the quality of care and reduces mortality and poor outcome [12–14]. Our audit followed by the intervention enabled the acquisition of symmetrical CT brain images with better diagnostic value. Presently there are many reliable but sophisticated methods that use intricate algorithms to correct asymmetry in CT brain images, but they are complex to use and require professional expertise [15, 16]. The advantage of using a simple and user friendly checklist while positioning the patient’s head on the CT table enables us to obtain symmetrical CT brain images in a quick and cost-effective manner.

## Lessons and limitations

We realized from this audit how a simple strategy like implementation of a checklist formulated from input of all stakeholders could improve the quality of a basic and vital investigation. The impeccable placement of landmark using laser points was vital in attaining symmetry. The simplicity of establishing anatomical landmarks present bilaterally to reach symmetry was the highlight of this project.

Despite the stringent application of checklist the improvement in symmetry accounted only for 30% patients. We could not prevent the novice, untrained residents entering the program being designated the job of accompanying patients to CT scan.

Although we were able to sustain the benefit of intervention in 3rd cycle, we do realize the need of repeated audits and frequent reemphasis on using landmarks to ascertain correct head positioning in CT gantry.

## Conclusion

The use of a straightforward and short checklist prior to CT brain in head injury helped acquire symmetrical scans of good diagnostic value. Moreover, sustainable improvement in reducing grossly asymmetrical scans following the intervention was also observed.

### Supplementary Information

**Additional file 1.**

## Data Availability

The datasets used and/or analysed during the current study are available from the corresponding author on reasonable request.

## References

[1] India State-Level Disease Burden Initiative Road Injury Collaborators (2020). Mortality due to road injuries in the States of India: the global burden of Disease Study 1990–2017. Lancet Public Health.

[2] Dewan MC, Rattani A, Gupta S, Baticulon RE, Hung Y-C, Punchak M (2018). Estimating the global incidence of traumatic brain injury. J Neurosurg.

[3] Carney N, Totten AM, O'Reilly C, Ullman JS, Hawryluk GW, Bell MJ, et al. Guidelines for the management of severe traumatic brain injury. 4th Ed. Neurosurgery. 2017;80:6–15. 10.1227/NEU.0000000000001432. 10.1227/NEU.0000000000001432 27654000

[4] Moore EE, Feliciano DV, Mattox KL. Trauma. 5th ed. McGraw-Hill Professional; 2003.

[5] Prima S, Ourselin S, Ayache N (2002). Computation of the mid-sagittal plane in 3-D brain images. IEEE Trans Med Imaging.

[6] Downer JJ, Pretorius PM (2009). Symmetry in computed tomography of the brain: the pitfalls. Clin Radiol.

[7] Liu Y, Collins RT, Rothfus WE (2001). Robust midsagittal plane extraction from normal and pathological 3-D neuroradiology images. IEEE Trans Med Imaging.

[8] Liu X, Imielinska C, Laine A, Connolly ES, D’Ambrosio AL. Symmetry Identification Using Partial Surface Matching and Tilt Correction in 3D Brain Images. In: 2006 International Conference of the IEEE Engineering in Medicine and Biology Society. 2006. p. 1056–60. 10.1109/IEMBS.2006.260642 17946874

[9] Harel Z, Silver SA, McQuillan RF, Weizman AV, Thomas A, Chertow GM, Harel Z, Silver SA, McQuillan RF, Weizman AV, Thomas A, Chertow GM, Nesrallah G, Chan CT, Bell CM (2016). How to diagnose solutions to a quality of Care Problem. Clin J Am Soc Nephrol.

[10] Winters BD, Gurses AP, Lehmann H, Sexton JB, Rampersad CJ, Pronovost PJ (2009). Clinical review: checklists - translating evidence into practice. Crit Care.

[11] Thomassen Ø, Espeland A, Søfteland E, Lossius HM, Heltne JK, Brattebø G (2011). Implementation of checklists in health care; learning from high-reliability organisations. Scand J Trauma Resusc Emerg Med.

[12] Verdaasdonk EGG, Stassen LPS, Hoffmann WF, van der Elst M, Dankelman J (2008). Can a structured checklist prevent problems with laparoscopic equipment?. Surg Endosc.

[13] Pronovost P, Needham D, Berenholtz S, Sinopoli D, Chu H, Cosgrove S, Pronovost P, Needham D, Berenholtz S, Sinopoli D, Chu H, Cosgrove S, Sexton B, Hyzy R, Welsh R, Roth G, Bander J, Kepros J, Goeschel C (2006). An intervention to decrease catheter-related bloodstream Infections in the ICU. N Engl J Med.

[14] Haynes AB, Weiser TG, Berry WR, Lipsitz SR, Breizat AHS, Dellinger EP (2009). A surgical safety checklist to reduce morbidity and mortality in a global population. N Engl J Med.

[15] Hu Q, Nowinski WL (2003). A rapid algorithm for robust and automatic extraction of the midsagittal plane of the human cerebrum from neuroimages based on local symmetry and outlier removal. NeuroImage.

[16] Chen W, Belle A, Cockrell C, Ward KR, Najarian K (2013). Automated midline shift and intracranial pressure estimation based on brain CT images. J Vis Exp.

