# Supplementary material for: Symmetry of computerised tomography of the brain in traumatic brain injury: a quality improvement audit
Source: BMC Neurol. 2023 Oct 31;23:391. doi: 10.1186/s12883-023-03441-x (PMC10617095; doi:10.1186/s12883-023-03441-x)
Supplement: Supplementary file 1 — Additional file 1. [file 12883_2023_3441_MOESM1_ESM.pdf]

Supplementary Material:

Checklist for CT brain study

Ensure Horizontal and Vertical Laser marker lines of the CT machine intersect at the Glabella.	☒
Horizontal marker line should superimpose on the bilateral supraorbital ridge, and the vertical marker line should be in the midline, and both the lines should intersect.	☐
Patient should be immobile.	☐
Ensure safe and adequate sedation if needed.	☐
